# Lipoprotein concentration in patients requiring extracorporeal membrane oxygenation

**DOI:** 10.1038/s41598-021-96728-3

**Published:** 2021-08-26

**Authors:** Sébastien Tanaka, Christian De Tymowski, Nathalie Zappella, Aurélie Snauwaert, Tiphaine Robert, Brice Lortat-Jacob, Yves Castier, Alexy Tran-Dinh, Parvine Tashk, Donia Bouzid, Marylou Para, Quentin Pellenc, Enora Atchade, Olivier Meilhac, Philippe Montravers

**Affiliations:** 1 Assistance Publique - Hôpitaux de Paris (AP-HP), Department of Anesthesiology and Critical Care Medicine, Bichat-Claude Bernard Hospital, Paris, France; 2 Réunion Island University, French Institute of Health and Medical Research (INSERM), U1188 Diabetes Atherothrombosis Réunion Indian Ocean (DéTROI), CYROI Plateform, Saint-Denis de La Réunion, France; 3 Center for Research on Inflammation, French Institute of Health and Medical Research (INSERM) U1149, Paris, France; 4 Université de Paris, UFR Paris Nord, Paris, France; 5 Assistance Publique - Hôpitaux de Paris (AP-HP), Biochemistry Department, Bichat-Claude Bernard Hospital, Paris, France; 6 Assistance Publique - Hôpitaux de Paris (AP-HP), Vascular and Thoracic Surgery Department, Bichat-Claude Bernard Hospital, Paris, France; 7 French Institute of Health and Medical Research (INSERM) U1148, Laboratory for Vascular Translational Science, Paris, France; 8 Assistance Publique - Hôpitaux de Paris (AP-HP), Emergency Department, Bichat-Claude Bernard Hospital, Paris, France; 9 French Institute of Health and Medical Research (INSERM) U1137, Infection, Antimicrobials, Modelling, Evolution, Paris, France; 10 Assistance Publique - Hôpitaux de Paris (AP-HP), Department of Cardiac Surgery, Bichat- Claude Bernard Hospital, Paris, France; 11 Réunion Island University-Affiliated Hospital, Saint-Denis de la Réunion, France; 12 French Institute of Health and Medical Research (INSERM) U1152, ANR-10-LABX-17, Physiopathology and Epidemiology of Respiratory Diseases, Paris, France

**Keywords:** Medical research, Cardiac device therapy, Endocrinology, Endocrine system and metabolic diseases

## Abstract

Extracorporeal membrane oxygenation (ECMO), a relevant technology for patients with acute respiratory distress syndrome (ARDS) or acute cardiac failure (ACF), is a frequent cause of systemic inflammatory response syndrome. During sepsis, HDL cholesterol (HDL-C) and LDL cholesterol (LDL-C) concentrations decrease, and an association between low lipoprotein levels and poor outcomes was reported. There are no data from patients undergoing ECMO. The goal of this study was to characterize the lipoprotein profiles of ICU patients requiring ECMO. All consecutive patients admitted for ARDS or ACF requiring ECMO were prospectively included. Daily lipoprotein levels and short-term prognosis outcome were assessed. 25 patients were included. On admission, lipoprotein concentrations were low, under the reference values ([HDL-C] = 0.6[0.4–0.8]mmol/L;[LDL-C] = 1.3[1.0–1.7]mmol/L). A statistically significant rise in lipoproteins overtime was observed during the ICU stay. We found no relationship between lipoproteins concentrations and mortality on Day-28 (*p* = 0.689 and *p* = 0.979, respectively). Comparison of surviving patients with non-surviving patients did not reveal any differences in lipoproteins concentrations. Stratification between septic and non-septic patients demonstrated that septic patients had lower lipoproteins concentrations on admission (HDL-C: 0.5[0.3–0.6]mmol/l vs 0.8[0.6–0.9]mmol/l, *p* = 0.003; LDL-C: 1.1[0.9–1.5]mmol/l vs 1.5[1.3–2.6]mmol/l; *p* = 0.012), whereas these two groups were comparable in terms of severity and outcomes. HDL-C concentrations during ICU hospitalization were also significantly lower in the septic group than in the non-septic group (*p* = 0.035). In conclusion, Lipoprotein concentrations are low in patients requiring ECMO but are not associated with poor outcomes. The subpopulation of septic patients had lower lipoprotein levels overtime, which reinforces the potential key-role of these particles during sepsis.

## Introduction

High-density lipoproteins (HDLs) are a family of nanoparticles characterized by their ability to transport cholesterol from peripheral tissues back to the liver, conferring a cardiovascular protective effect^1,2^. In addition to this function, HDLs display pleiotropic properties, including anti-inflammatory, antioxidant, antiapoptotic or anti-infectious functions^3–5^. To take advantage of this global endothelial protective effect, several experimental and clinical studies have been conducted in inflammatory conditions^5,6^. During sepsis, which represents an exacerbated state of acute inflammation particularly affecting the endothelium, clinical studies have reported decreased HDL levels during the acute phase and an association between low levels of HDL cholesterol (HDL-C) and poor outcomes^7–11^. In addition to a quantitative decrease in particles, qualitative modifications leading to HDL dysfunction during sepsis have been described^12–17^. In this context, supplementation with functional reconstituted HDLs or mimetic peptides has been performed in animal models of sepsis and demonstrated a beneficial effect on inflammatory markers and on morbi-mortality^18–21^. Studies involving inflammatory non-septic patients have reported more controversial results. Chenaud et al. observed decreased levels of apolipoprotein-A1 (the major HDL protein) in patients hospitalized in the intensive care unit (ICU) for systemic inflammatory response syndrome (SIRS)^22^. In a previous study, we compared HDL profiles between two populations with exacerbated acute inflammation, sepsis and trauma, and the results revealed that HDL-C concentrations were low in septic patients but not in cases of trauma^23^.

Extracorporeal life support (ECLS), including cardiopulmonary bypass (CPB) and extracorporeal membrane oxygenation (ECMO), is a relevant technology with an exponential expansion worldwide, increasing improvement in outcomes for patients with respiratory or cardiac failure^24,25^. However, ECMO is a frequent cause of systemic inflammation^26^. Exposition of blood to the extracorporeal circuit during ECMO induces an inflammatory response that can mimic SIRS^26,27^.

To the best of our knowledge, lipoproteins and especially HDL concentrations have never been investigated in patients with an inflammatory state induced by ECMO. The goal of the present study was to determine lipid levels over time in ICU patients requiring ECMO and to characterize the potential links between these lipoproteins and patient outcomes. The second goal was to stratify the patients according to the presence or absence of sepsis to compare these two acute inflammatory entities.

## Materials and methods

This was a monocentric study conducted in the surgical ICU of Bichat Claude-Bernard University Hospital, Paris, France. Patients admitted from December 2019 to April 2020 for acute respiratory distress syndrome (ARDS) or acute cardiac failure (ACF) requiring ECMO support were consecutively and prospectively included in a database, and their medical charts were reviewed retrospectively. The study was approved by the French Society of Anesthesiology and Critical Care Medicine Research Ethics Board, which waived an informed consent (ECMOLIPID study, IRB 00,010,254-2020-119).

Patient demographics, Simplified Acute Physiology Score II (SAPSII), Sepsis-related Organ Failure Assessment (SOFA) severity scores and clinical data were collected. Mortality at 28 days (Day-28), duration of mechanical ventilation, number of days alive without mechanical ventilation at Day-28, length of stay in the ICU and in the hospital, renal replacement therapy, vasopressor use, and tracheostomy were collected. The type of device (veno-venous ECMO (VV-ECMO) or veno-arterial ECMO (VA-ECMO)) and its duration were also documented. As soon as ECMO was started, all patients received a continuous intravenous infusion of unfractionated heparin for the duration of ECMO support.

Lipoprotein concentration appears to be associated with patient prognosis and could be used as a sepsis biomarker^5,7,28^. In this context, we routinely purposely determined the plasma lipid profile (total cholesterol (TC), HDL-C, LDL-C, triglycerides (TG)) for all our ICU patients. Lipid concentrations were thus collected after ECMO cannulation and daily until its removal. Lipid tests were performed in the Biochemistry Laboratory of Bichat Claude-Bernard Hospital. Total cholesterol (TC), HDL-C, LDL-C and triglyceride concentrations were determined by routine enzymatic assays (CHOL, HDLC, LDLC and TRIG methods, Dimension VISTA System, Siemens Healthineers). The reference values for these assays were HDL-C: > 1.40 mmol/L; total cholesterol (TC): 4.40 < N < 5.20 mmol/L and triglycerides: 0.50 < N < 1.7 mmol/L. According to the French National Authority for Health 2017 and the European Society of Cardiology 2016 recommendations, LDL-C concentration targets have been established depending on vascular risk factors^29^. According to these recommendations, LDL-C < 3.0 mmol/L and LDL-C < 2.6 mmol/L concentrations are advised in low to moderate and high cardiovascular risk patients, respectively. All methods were carried out in accordance with guidelines.

Patients were also stratified according to the presence or absence of sepsis at the time of ICU hospitalization. The diagnosis of sepsis was performed according to the Third International Consensus Definitions for Sepsis and Septic Shock (Sepsis-3)^30^. Data relative to sepsis were also compiled.

### Statistical analysis

Continuous variables are expressed as medians with interquartile ranges (IQRs) and were compared with the Mann–Whitney U test. Categorical variables were expressed as counts and percentages and were compared with Fisher’s exact test or the chi-square test. The threshold defining the lower quartile was determined to have 25% of the overall population in that quartile. Correlations were assessed by Spearman’s rank order correlation. Survival analyses were estimated with Kaplan–Meier analyses and compared by the log-rank test. A mixed model for repeated measures was performed to compare the evolution of lipoprotein concentrations over according to day-28 mortality. A *p* value < 0.05 was considered statistically significant. All statistical analyses were performed using SPSS software, version 21 (IBM, Armonk, NY USA).

### Ethical statement

The study was approved by the French Society of Anesthesiology and Critical Care Medicine Research Ethics Board (ECMOLIPID study, IRB 00,010,254-2020-119).

## Results

### Population

Overall, 25 patients were hospitalized in our ICU for ARDS (n = 18, 72%) or ACF (n = 7, 28%) and required ECMO (VV-ECMO: n = 17; VA-ECMO: n = 8). Ninety-six percent of patients underwent ECMO implantation on the day of ICU admission (median day = 1[1]). The median ECMO duration was 5 [2–12] days. The general characteristics of the overall population are presented in Table 1.

**Table 1:** General characteristics and outcomes of the patients.

Characteristics	Overall population (n = 25; 100%)	Alive day-28 (n = 13; 52%)	Dead day-28 (n = 12; 48%)	*p* value
Age, years	53 [42–59]	46 [34–54]	55 [52–59]	0.168
Male sex	16 (64)	9 (69)	7 (58)	0.688
Weight (kg)	73 [50–89]	73 [54–86]	83 [46–92]	0.833
Length (cm)	172 [164–175]	173 [171–178]	168 [162–175]	0.228
BMI, kg/m^2^	25 [19–28]	25 [21–28]	27 [18–31]	0.722
**Presence of comorbidities**				
High blood pressure	7 (28)	4 (31)	3 (25)	1
Diabetes mellitus	3 (12)	1 (8)	2 (17)	0.593
Statin	3 (12)	2 (15)	1 (8)	1
**Severity scores**				
SAPSII on admission	55 [44–70]	55 [33–57]	67 [51–76]	0.046
SOFA on admission	8 [6–11]	8 [6–8]	11 [6–12]	0.168
**Treatments during ICU stay**				
Norepinephrine	23 (92)	13 (100)	10 (835)	0.220
Length of mechanical ventilation	17 [5–28]	26 [18–43]	6 [3–11]	0.001
Days without mechanical ventilation	0 [0–11]	11 [5–15]	0 [0–0]	< 0.001
Tracheostomy	10 (40)	10 (77)	0 (0)	< 0.001
RRT	12 (48)	5 (39)	7 (58)	0.434
Veno-venous ECMO	17 (68)	7 (54)	10 (83)	0.202
Veno-arterial ECMO	8 (32)	6 (46)	2 (17)	0.202
**Outcomes**				
Sepsis	15 (60)	7 (54)	8 (67)	0.688
Bacteremia	1 (4)	0 (0)	1 (8)	1
VAP	16 (64)	10 (77)	6 (50)	0.226
Number of VAP	0 [1, 2]	2 [1–3]	0 [0–1]	0.052
ICU LOS	23 [7–40]	40 [25–53]	7 [3–12]	< 0.001
Hospital LOS	26 [7–60]	64 [46–112]	7 [3–12]	< 0.001

Continuous variables are expressed as the median and interquartile range (IQR) and were compared using the Mann–Whitney U test. Categorical variables are expressed as n (%) and were compared with Fisher’s exact test; BMI: body mass index; ECMO: extracorporeal membrane oxygenation; LOS: length of Stay; RRT: renal replacement therapy; SAPS II: simplified acute physiology score II; SOFA: sepsis-related organ failure; VAP: ventilator-associated pneumonia.

### Lipid profile on admission and kinetics over time

On admission, TC, TG, HDL-C and LDL-C concentrations were 2.4 [2.0–3.6] mmol/L, 1.9 [0.9–2.8] mmol/L, 0.6 [0.4–0.8] mmol/L and 1.3 [1.0–1.7] mmol/L, respectively. Except TG, all other concentrations were under the abovementioned reference values (see "Materials and methods" section).

**Figure 1:**
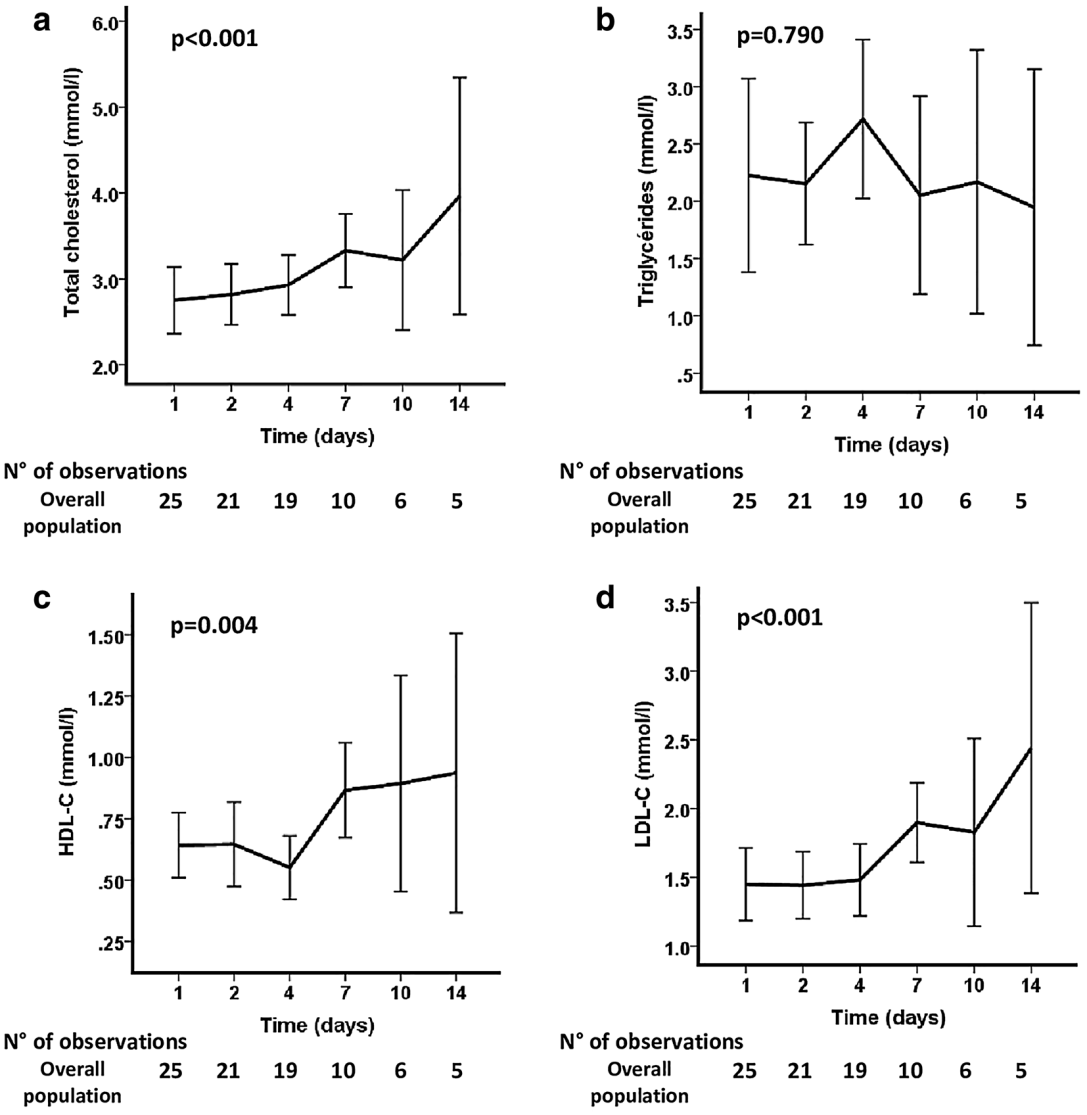
Mean (95% CI) variations of lipoprotein concentrations during ICU stay for the overall population. Time comparison (*p* value) was performed using a mixed model. (**a**) Total Cholesterol (TC), (**b**) Triglycerides (TG), (**c**) High-density lipoproteins cholesterol (HDL-C) and (**d**) Low-density lipoproteins cholesterol (LDL-C) concentrations throughout the ICU stay.

Figure 1 represents the kinetics of TC, TG, HDL-C and LDL-C concentrations throughout the ICU stay. Except for the TG concentration, which remained in the normal range of reference values, we observed a statistically significant progressive increase in TC, HDL-C and LDL-C during the study period (*p* = 0.001, *p* = 0.004 and *p* < 0.001, respectively), which had returned to normal values by the end of the study in the survivors.

To better characterize these variations, we stratified lipoprotein values according to the fluid balance calculated as the difference between fluid intake and output. The median fluid balance at Day-1 of 1300 ml was selected as cut-off value. We found no statistically significant difference between the two groups concerning TC, HDL-C, LDL-C and triglycerides values during the first seven days following admission (see supplemental Fig. S1).

### Relationship between lipid profile and mortality

**Figure 2:**
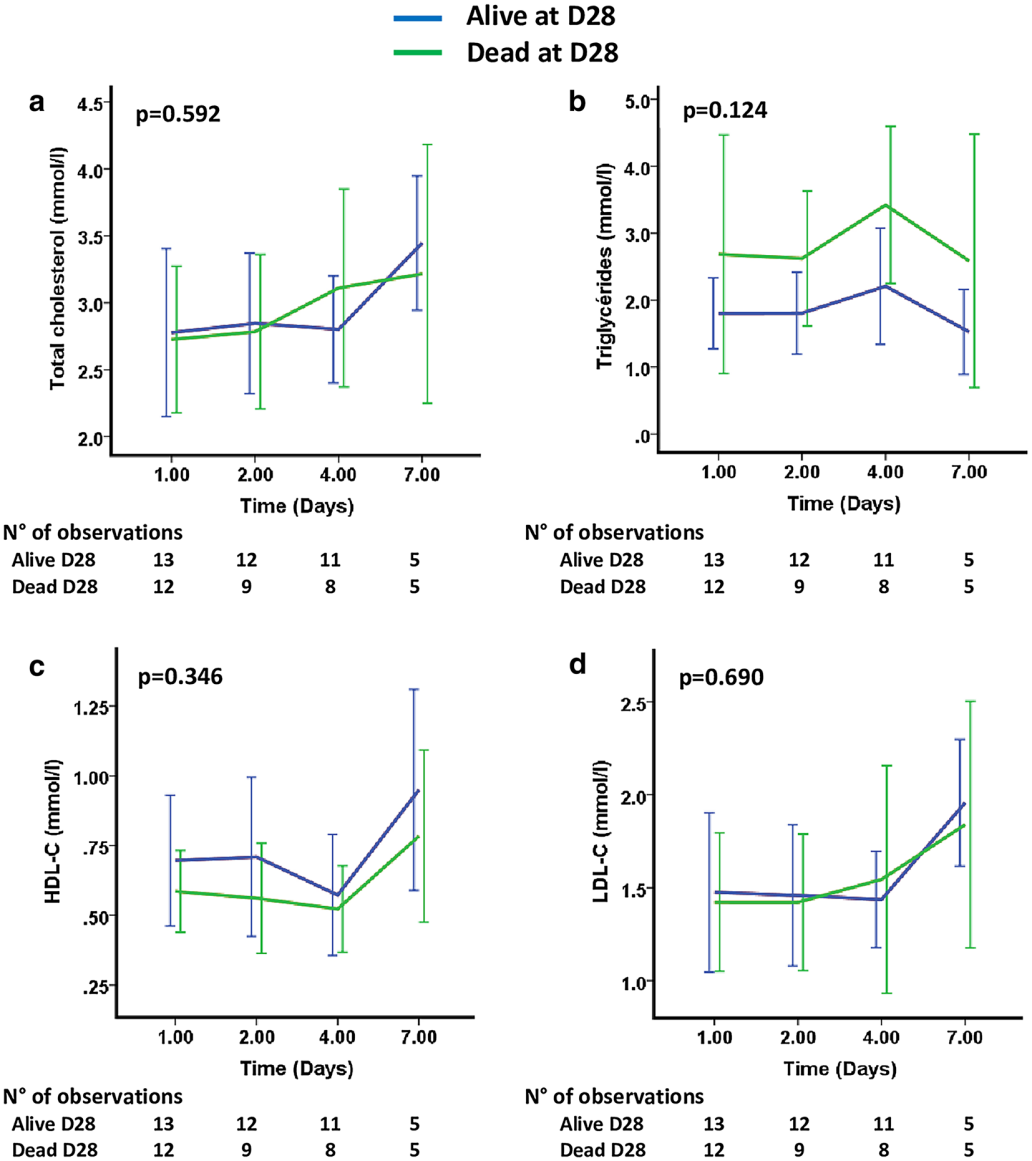
Mean (95% CI) variations of lipoprotein concentrations during the first seven days following admission according to Day-28 mortality. Group comparison (*p* value) was performed using a mixed model. (**a**) Total Cholesterol (TC), (**b**) Triglycerides (TG), (**c**) High-density lipoproteins cholesterol (HDL-C) and (**d**) Low-density lipoproteins cholesterol (LDL-C) concentrations throughout the ICU stay.

On ICU admission, there was no difference in lipid profiles between the surviving and non-surviving patients (TC: 2.4 [2.0–3.8] mmol/L vs. 2.4 [2.4–3.3] mmol/L, *p* = 0.894; TG: 1.5 [1.1–2.8] mmol/L vs. 2.1 [0.9–3.2] mmol/L; *p* = 0.769; HDL-C: 0.6 [0.4–0.9] mmol/L vs. 0.6 [0.4–0.8] mmol/L, *p* = 0.689; LDL-C: 1.4 [0.9–2.0] mmol/L vs. 1.3 [1.1–1.5] mmol/L, *p* = 0.979). In addition, changes in the TC, TG, HDL-C and LDL-C concentrations over time during the first seven days following admission did not allow us to distinguish survivors from nonsurvivors at Day-28 (Fig. 2).

**Figure 3:**
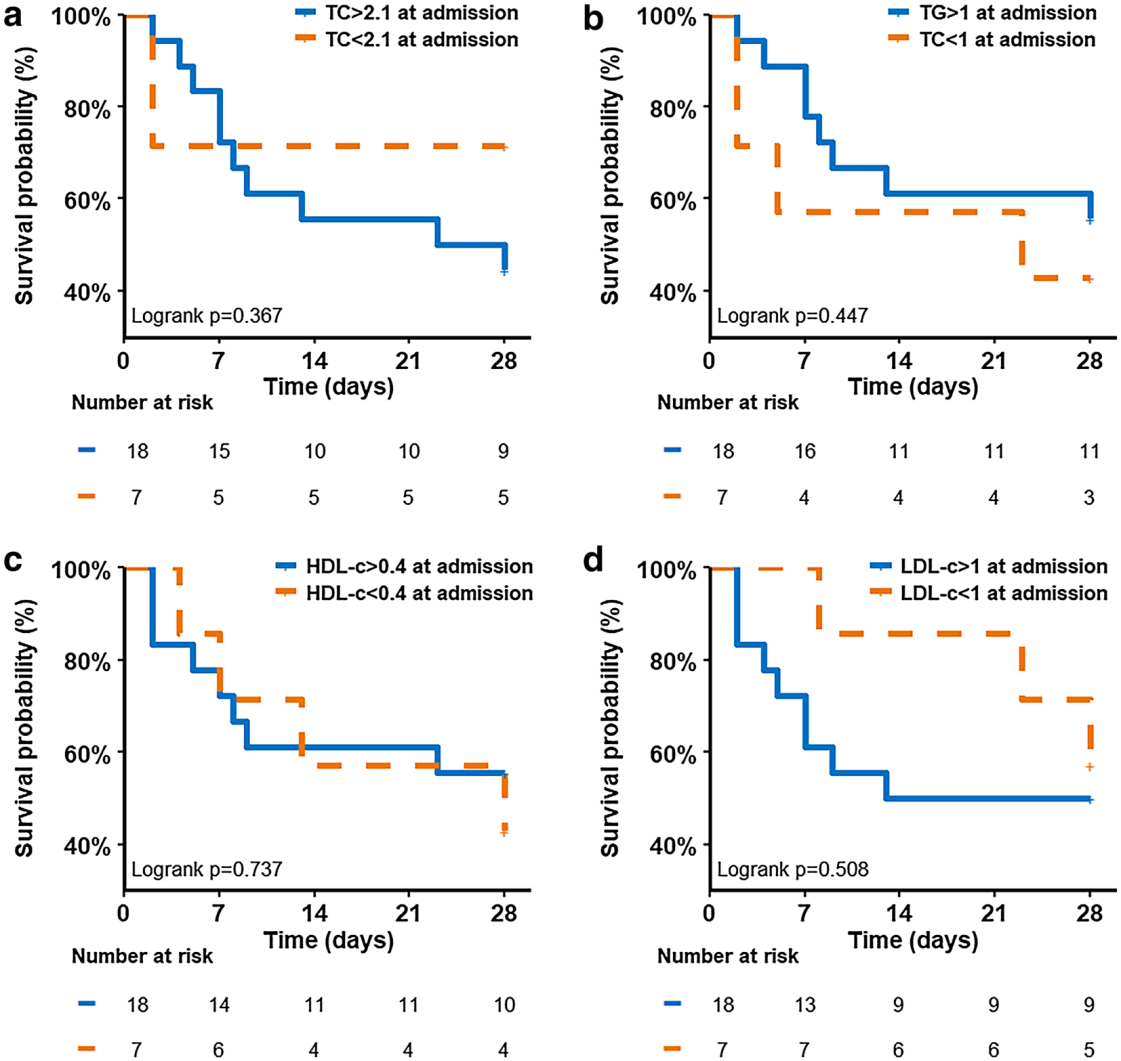
Mortality at day 28 according to the lipid value at admission estimated with Kaplan–Meier analyses and compared by the log-rank test. (**a**) Total Cholesterol (TC). The value of 2.1 mmol/l represents the cut-off to define the first quartile of patient according to TC at admission (< 2.1 mmol /l). (**b**) Triglycerides (TG). The value of 1 mmol/l represents the cut-off to define the first quartile of patient according to TG at admission (< 1 mmol /l). (**c**) High-density lipoproteins cholesterol (HDL-C). The value of 0.4 mmol/l represents the cut off to define the first quartile of patient according to HDL-c at admission (< 0.4 mmol /l) and (**d**) Low-density lipoproteins cholesterol (LDL-C). The value of 1 mmol/l represents the cut-off to define the first quartile of patient according to LDL-C at admission (< 1 mmol /l).

Figure 3 represents mortality at Day-28 according to the lipid profile on admission estimated with Kaplan–Meier analyses and compared by the log-rank test. No relationship was found between patients with TC, TG, HDL-C and LDL-C concentrations in their respective lower quartile and mortality at Day-28 (log rank *p* = 0.367, *p* = 0.447, *p* = 0.737 and *p* = 0.508, respectively).

### Relationship between the lipid profile and type of ECMO device and patient outcome

No differences in TC and LDL-C on admission were found between patients receiving VA-ECMO and those receiving VV-ECMO (TC: 2.5 [2.0–3.5] mmol/L for VA-ECMO and 2.4 [2.1–3.4] mmol/L for VV-ECMO, p = 0.887; LDL-C: 1.4 [1.1–2] mmol/L for VA-ECMO and 1.3 [1–1.60] mmol/L for VV-ECMO, p = 0.475). Patients receiving VV-ECMO had higher TG concentrations and lower HDL-C concentrations on admission than those receiving VA-ECMO (2.3 [1.3–2.8] mmol/L vs 1.0 [0.8–1.4] mmol/l, *p* = 0.031 and 0.5 [0.3–0.6] mmol/L vs 0.8 [0.7–1.0] mmol/L, *p* = 0.005, respectively).

In addition, TG concentrations over time during the first seven days following admission were significantly lower in VV-ECMO patients than in VA-ECMO patients (*p* = 0.037, Supplemental Fig. S1). Moreover, no differences were found between VV-ECMO and VA-ECMO patients in terms of changes in TC, HDL-C and LDL-C concentrations over time during the first seven days following admission (Supplemental Fig. S2).

Table 2 shows the comparison of the relationship between the TC, TG, HDL-C and LDL-C concentrations on admission and ICU outcome (renal replacement therapy, need for norepinephrine). No statistical link was found between TC, TG, HDL-C and LDL-C on admission and these outcome parameters.

**Table 2:** Relationship between lipid profile on admission and outcome variables.

Biology on admission	Overall population (n = 25)	No RTT (n = 13; 52%)	RTT (n = 12; 48%)	*P* value
Total cholesterol	2.4 [2.1–3.6]	2.2 [2.1–3.2]	2.5 [2.0–3.8]	0.728
Triglyceride	1.9 [1.0–2.8]	1.4 [1.1–2.6]	2.1 [0.9–3.0]	0.503
HDL-c	0.6 [0.4–0.8]	0.6 [0.5–1.0]	0.5 [0.3–0.7]	0.376
LDL-C	1.3 [1.0–1.7]	1.1 [1.0–1.6]	1.4 [1.1–1.7]	0.295

Biology on admission	Overall population (n = 26)	No NOR (n = 2; 7%)	NOR (n = 24; 93%)	*P* value
Total cholesterol	2.4 [2.0–3.6]	2.8 [2.2–3.4]	2.4 [2.1–3.5]	0.807
Triglyceride	1.9 [0.9–2.8]	3.2 [2.6–3.8]	1.5 [0.9–2.8]	0.167
HDL-c	0.6 [0.4–0.8]	0.5 [0.3–0.7]	0.6 [0.5–0.8]	0.480
LDL-C	1.3 [1.0–1.7]	1.3 [1.1–1.6]	1.3 [1.0–1.7]	0.960

Variables: renal replacement therapy and need for norepinephrine. Continuous variables are expressed as the median and interquartile range (IQR). RRT: renal replacement therapy; NOR: norepinephrine.

We did not observe any correlation between the TC, TG, HDL-C and LDL-C concentrations collected on the day of ECMO implantation and ICU length of stay or the number of days alive at Day-28 without mechanical ventilation. However, HDL-C and LDL-C concentrations on the day of ECMO implantation were inversely correlated with ECMO duration (HDL-C: Rho of Spearman = − 0.455, *p* = 0.022; LDL-C: Rho of Spearman = − 0.535, *p* = 0.006) (Supplemental Table S3).

### Stratification between septic and non-septic patients

At the time of ICU hospitalization, 15 patients had sepsis. Nine patients had sepsis due to COVID-19 pneumonia, 1 *Pseudomonas aeruginosa* pneumonia, 3 *Staphylococcus aureus* pneumonia, 1 *Achromobacter xylosoxidans* pneumonia and 1 *Pseudomonas aeruginosa* mediastinitis.

The general characteristics of the patients with and without sepsis are presented in Table 3.

**Table 3:** General characteristics and outcome of the patients with and without sepsis.

Characteristics	Overall population (n = 25; 100%)	Without sepsis (n = 10; 40%)	Sepsis (n = 15; 60%)	*P* value
Age. years	53 [42–59]	54 [37–62]	52 [43–57]	0.461
Male sex	16 (64)	5 (50)	11 (73)	0.222
Weight (kg)	73 [50–89]	71 [56–84]	85 [49–94]	0.456
Length (cm)	172 [164–175]	172 [164–173]	173 [164–180]	0.771
BMI. kg/m^2^	25 [19–28]	25 [19–27]	27 [19–32]	0.418
**Presence of comorbidities**				
High blood pressure	7 (28)	2 (20)	5(33)	0.467
Diabetes mellitus	3 (12)	0 (0)	3 (20)	0.250
Statin therapy	3 (12)	1 (10)	2 (13)	1
**Severity scores**				
SAPSII	55 [44–70]	58 [29–76]	55 [46–62]	0.978
SOFA	8 [6–11]	9 [4–13]	8 [7–10]	0.605
**Treatments during ICU stay**				
Norepinephrine	23 (92)	10 (100)	13 (87)	0.500
Length of mechanical ventilation	17 [5–28]	19 [2–43]	17 [7–26]	0.978
Days without mechanical ventilation	0 [0–11]	0 [0–11]	0 [0–8]	0.907
Tracheotomy	10 (40)	4 (40)	6 (40)	1
RRT	12 (48)	6 (60)	6 (40)	0.428
Veno-venous ECMO	17 (68)	5 (50)	12 (80)	0.194
Veno-arterial ECMO	8 (32)	5 (50)	3 (20)	0.115
**Outcomes**				
Death at Day-28	12 (48)	4 (40)	8 (53)	0.688
Bacteremia	1 (4)	0 (0)	1 (7)	0.405
VAP	16 (64)	4 (40)	12 (80)	0.087
Number of VAP	0 [1, 2]	0 [0–2]	1 [1,2]	0.196
ICU LOS	23 [7–40]	22 [2–43]	23 [8–34]	0.640
Hospital LOS	26 [7–60]	21 [2–87]	23 [8–58]	0.570

Continuous variables are expressed as the median and interquartile range (IQR) and were compared using the Mann–Whitney U test. Categorical variables are expressed as n (%) and were compared with Fisher’s exact test; BMI: body mass index; ECMO: extracorporeal membrane oxygenation; LOS: length of Stay; RRT: renal replacement therapy; SAPS II: simplified acute physiology score II; SOFA: sepsis-related organ failure; VAP: ventilator-associated pneumonia.

Sepsis was reported in 12 (80%) patients receiving VV-ECMO and 3 (20%) patients receiving VA-ECMO (*p* = 0.193).

Septic patients had significantly lower HDL-C and LDL-C concentrations on admission than patients without sepsis (HDL-C: 0.5 [0.3–0.6] mmol/L vs 0.8 [0.6–0.9] mmol/L, *p* = 0.003; LDL-C: 1.1 [0.9–1.5] mmol/L vs 1.5 [1.3–2.6] mmol/L; *p* = 0.012, respectively).

Septic patients had higher TG concentrations on admission than non-septic patients (2.6 [1.4–3.0] mmol/L vs 1.0 [0.9–1.9] mmol/L; *p* = 0.031, respectively).

No significant difference was found between these two groups in terms of TC concentration on admission (2.3 [2.2–3.2] mmol/L vs 2.6 [1.9–3.8] mmol/L; *p* = 0.807, respectively).

**Figure 4:**
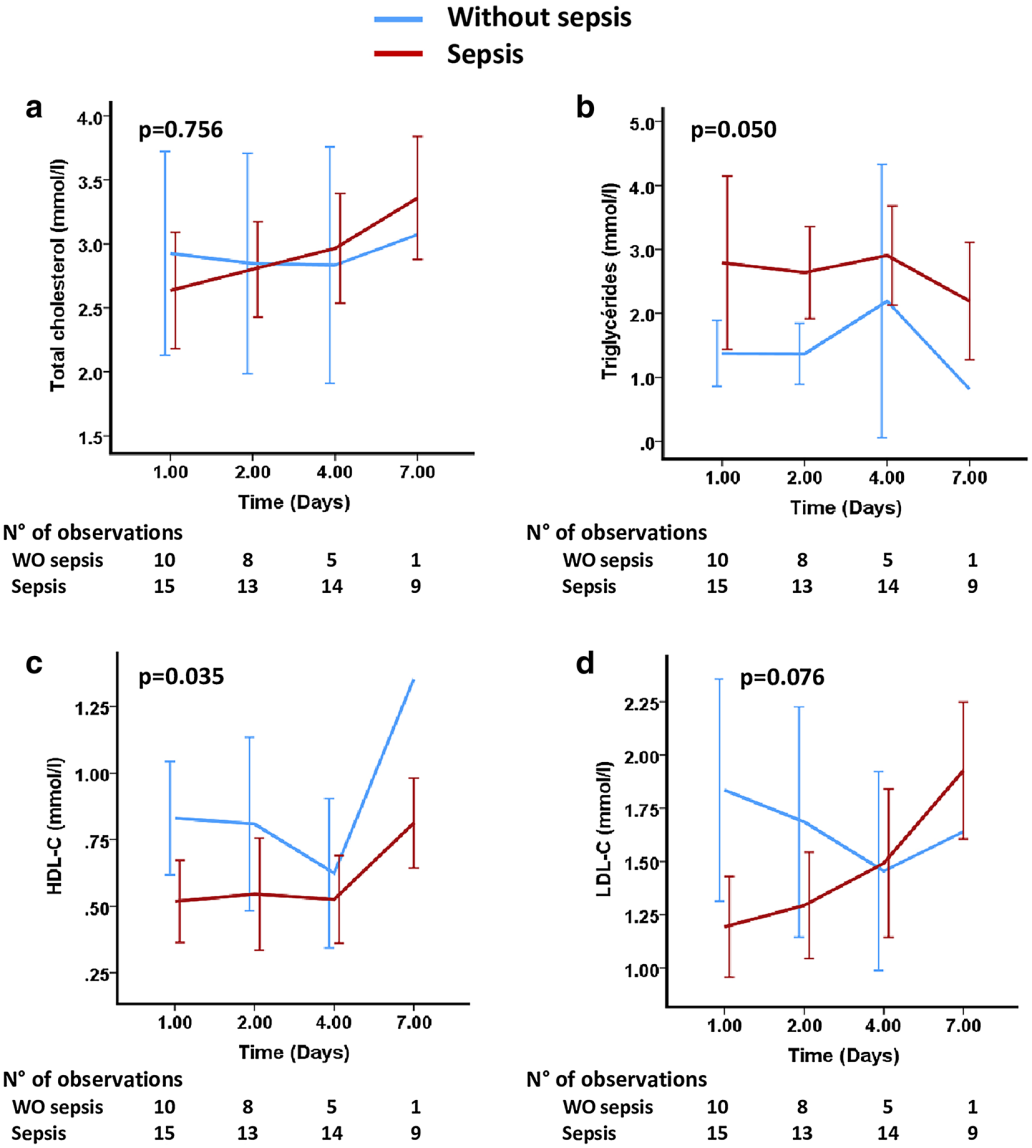
Mean (95% CI) variations of lipoprotein concentration during the first seven days following admission according to sepsis at admission. Group comparison (*p* value) was performed using a mixed model. Day 7 data in the group without sepsis is from only one patient. (**a**) Total Cholesterol (TC), (**b**) Triglycerides (TG), (**c**) High-density lipoproteins cholesterol (HDL-C) and (**d**) Low-density lipoproteins cholesterol (LDL-C) concentrations throughout the ICU stay.

In addition, HDL-C concentrations during the first seven days following admission were significantly lower in the septic group than in the non-septic group (*p* = 0.035, Fig. 4). Moreover, we did not observe any difference between septic and non-septic patients in terms of changes in TC, TG and LDL-C concentrations during the first seven days following admission (Fig. 4).

## Discussion

In our cohort of patients with ECMO device supportive therapy, we observed that lipoprotein concentrations at ICU admission were low but not statistically related to patient outcome. Interestingly, patients in the sepsis subgroup had significantly lower levels of lipoproteins on admission than patients in the non-sepsis subgroup. Specifically, the HDL-C concentration over time was lower in the septic group than in the non-septic group.

To the best of our knowledge, this is the first study assessing lipid profiles in ICU patients treated with ECMO. Few studies have reported lipid concentrations in patients undergoing cardiac surgery with cardiopulmonary bypass, which uses an extracorporeal circulation device. In the paper by Landymore et al. evaluating HDL-C and LDL-C concentrations after coronary arterial bypass requiring cardiopulmonary bypass, total serum cholesterol was decreased by 45%, while LDL-C and HDL-C levels were reduced by 50% and 30%, respectively^31^. Similarly, Hacquebard et al. observed changes in plasma LDL and HDL concentrations in 31 patients undergoing cardiac surgery^32^. Interestingly, beyond changes in certain lipid species (decreased cholesteryl esters and increased phospholipids), cardiac surgery induced substantial modifications in plasma apolipoproteins ApoB and ApoAI, the major proteins of LDL and HDL, respectively, with decreased concentrations noted under cardiopulmonary bypass. Associated with the decreased ApoAI concentration was an abrupt rise in serum amyloid A (SAA, an inflammatory protein) associated with HDL. Paraoxonase, an antioxidant enzyme associated with HDL particles, was also decreased. These changes indirectly reflect the proinflammatory state induced by extracorporeal circulation devices.

ECMO is a frequent cause of systemic inflammation, and exposure of the blood to the extracorporeal device may stimulate an inflammatory response that can mimic SIRS^26,27^. To explain the decrease in HDL-C and LDL-C in patients requiring ECMO, several hypotheses are possible, including acute overconsumption of HDL particles and a decrease in liver HDL synthesis (especially in the case of hepatic failure)^33^. HDL particles may also easily be redistributed from the intravascular to the extravascular compartment in this context. Although none of these mechanisms can be totally ruled out and deserve mechanistic studies, the part of hemodilution that could explain the decrease in lipoproteins and especially HDL particles does not seem to be predominant on the basis of our results. Finally, since HDLs are nanoparticles and the diameter of ECMO membranes is in the micrometer range, absorption of HDL particles onto the ECMO membrane seems unlikely. However, no studies have been conducted to investigate this possibility, especially in the case of dysfunctional HDL particles. In situ, pre- and post-membrane assays could be highly informative. Interestingly, unlike HDL-C and LDL-C concentrations, the level of TG remained approximately the same over time in our study. A possible explanation is that the prolonged systematic use of propofol, which thus increases the plasma triglyceride concentrations^34^, may be counterbalanced by the systematic use of unfractionated heparin. Indeed, unfractionated heparin leads to an increased synthesis of lipoprotein lipase, which increases the hydrolysis of TG from plasma lipoproteins such as very low density lipoproteins or chylomicrons^35^.

One major finding of this study is the marked difference observed in lipid profiles between ECMO patients with and without sepsis. Septic patients had lower HDL-C and LDL-C concentrations on admission, and HDL-C concentrations over time were lower in the septic group than in the non-septic group. This major difference between these two inflammatory conditions could be due to the interaction between lipoprotein and microorganism components. For example, one major action of HDL particles during sepsis is the clearance of bacterial components, such as lipopolysaccharides (LPS)^36,37^. After binding bacterial components, the clearance of HDL particles may also be increased in inflammatory conditions^38^. These observations reinforce the key role of lipoproteins during sepsis. Both the quantitative decrease in HDL particles and qualitative dysfunction of these lipoproteins observed during sepsis emphasize the potential positive effect of functional HDL particle supplementation during sepsis, which is an active field of investigation^5^.

This study has several limitations. First, it is a small monocentric study, which may partly explain the lack of association between lipoprotein concentration and morbi-mortality. We only have found a relationship between low concentrations of lipoprotein and a prolonged duration of ECMO. This observation is consistent with existing data on the association between lipoprotein concentrations and patient severity during inflammatory states. However, this observation has to be confirmed in larger cohorts of patients. Second, we did not measure inflammatory parameters, which could have been used to stratify the patients according to their inflammatory condition. Third, we did not compare lipid profiles on admission with basal concentrations prior to hospitalization, which could have been informative. In a recent work involving 206 septic patients, we did not find any link between lipoprotein concentrations prior to sepsis and the outcome^11^. Nevertheless, lipoproteins values before ECMO implantation in this particular population could be an important information to collect in a future high-powered study. Finally, we found a difference in HDL-C and LDL-C concentrations between veno-arterial and veno-venous ECMO devices that could have introduced bias. However, no study has yet described the difference in the inflammatory profile between the two types of ECMO^27^. Similarly, to our knowledge, no study ever identified a difference between cardiogenic shock and ARDS in terms of the lipid profile, and these pathologies are the main etiologies of these two types of ECMO devices (VA-ECMO and VV-ECMO, respectively).

In conclusion, this study compared for the first time the lipid profiles over time in patients requiring ECMO for ACF or ARDS and showed a decrease in lipoprotein levels but no association with patient outcomes. Moreover, the presence of sepsis could strongly differentiate patients with lower concentrations of HDL-C and LDL-C (in the case of sepsis), which underlines the major role of these lipoproteins in septic conditions. Further studies are needed to better characterize lipoprotein dysfunctions under these inflammatory states. Studies addressing other causes of inflammation, such as pancreatitis or burns, could also aid in clarifying the role played by lipoprotein in these different types of disease.

## Supplementary Information

Supplementary Information.

## Data Availability

All relevant data are within the manuscript and its Supporting Information files.
