# Supplementary material for: Lipoprotein concentration in patients requiring extracorporeal membrane oxygenation
Source: Sci Rep. 2021 Aug 26;11:17225. doi: 10.1038/s41598-021-96728-3 (PMC8390666; doi:10.1038/s41598-021-96728-3)
Supplement: Supplementary file 1 — Supplementary Information. [file 41598_2021_96728_MOESM1_ESM.pdf]

Lipoprotein concentration in patients requiring extracorporeal membrane oxygenation, results from the ECMOLIPID study

Sébastien TANAKA[1,2*], Christian DE TYMOWSKI[1,3,4], Nathalie ZAPPELLA[1], Aurélie SNAUWAERT[1], Tiphaine ROBERT[5], Brice LORTAT-JACOB[1], Yves CASTIER[4,6,7], Alexy TRAN-DINH[1,4,7], Parvine TASHK[1], Donia BOUZID[4,8,9], Marylou PARA[4,7,10], Quentin PELLENC[6,7], Enora ATCHADE[1], Olivier MEILHAC[2,11†], Philippe MONTRAVERS[1,4,12†]

1- Assistance Publique - Hôpitaux de Paris (AP-HP), Department of Anesthesiology and Critical Care Medicine, Bichat-Claude Bernard Hospital, Paris, France

2- Réunion Island University, French Institute of Health and Medical Research (INSERM), U1188 Diabetes atherothrombosis Réunion Indian Ocean (DéTROI), CYROI Plateform, Saint-Denis de La Réunion

3- French Institute of Health and Medical Research (INSERM) U1149, Center for Research on Inflammation, Paris, France

4- Université de Paris, UFR Paris Nord, Paris, France

5- Assistance Publique - Hôpitaux de Paris (AP-HP), Biochemistry Department, Bichat-Claude Bernard Hospital, Paris, France

6- Assistance Publique - Hôpitaux de Paris (AP-HP), Vascular and Thoracic Surgery Department, Bichat-Claude Bernard Hospital, Paris, France

7- French Institute of Health and Medical Research (INSERM) U1148, Laboratory for Vascular Translational Science, Paris France

8- Assistance Publique - Hôpitaux de Paris (AP-HP), Emergency Department, Bichat-Claude Bernard Hospital, Paris, France

9- French Institute of Health and Medical Research (INSERM) U1137, Infection, Antimicrobials, Modelling, Evolution, Paris, France

10- Assistance Publique - Hôpitaux de Paris (AP-HP), Department of Cardiac Surgery, Bichat- Claude Bernard Hospital, Paris, France

11- Réunion Island University-affiliated Hospital, France

12- French Institute of Health and Medical Research (INSERM) U1152, ANR-10-LABX-17, Physiopathology and Epidemiology of respiratory diseases, Paris, France

Supplemental figure S1 : lipoprotein concentrations during the first seven days following admission according to the fluid balance at Day-1 (cut-off = 1300 ml at Day 1).

a

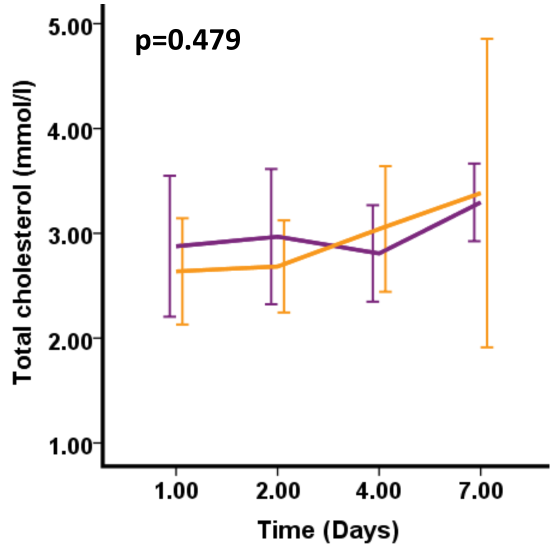

N° of observations

FB ≤1300 ml	12	10	9	6
FB >1300 ml	13	11	10	4

b

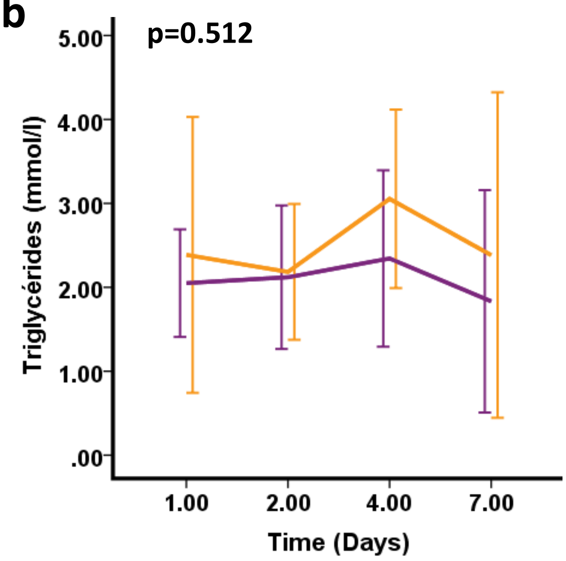

N° of observations

FB ≤1300 ml	12	10	9	6
FB >1300 ml	13	11	10	4

c

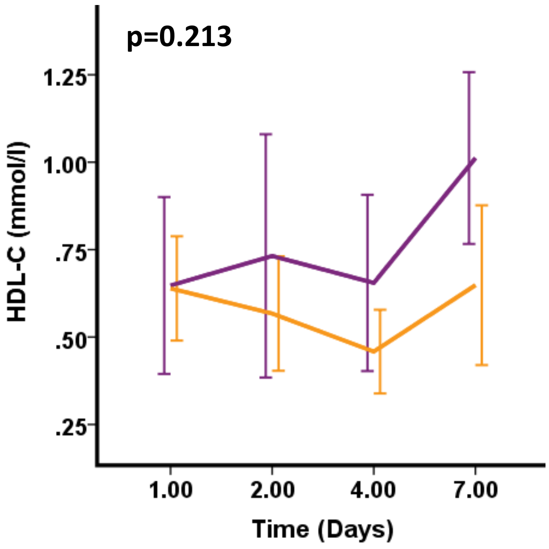

N° of observations

FB ≤1300 ml	12	10	9	6
FB >1300 ml	13	11	10	4

d

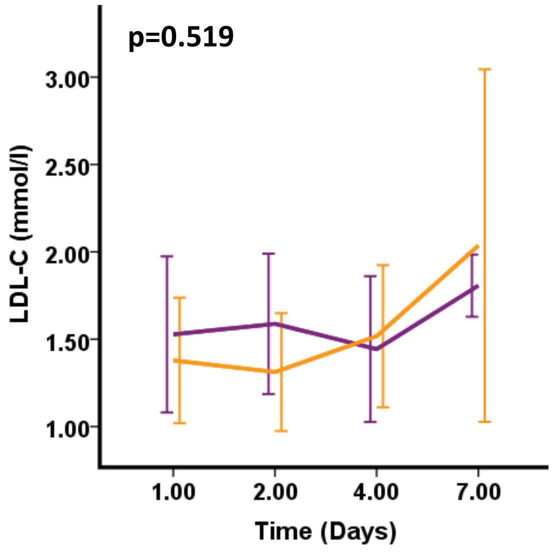

N° of observations

FB ≤1300 ml	12	10	9	6
FB >1300 ml	13	11	10	4

Fluid balance (FB) ≤1300 ml
Fluid balance (FB) >1300 ml

Supplemental figure S2 : lipoprotein concentrations during the first seven days following admission between VV-ECMO patients and VA-ECMO patients.

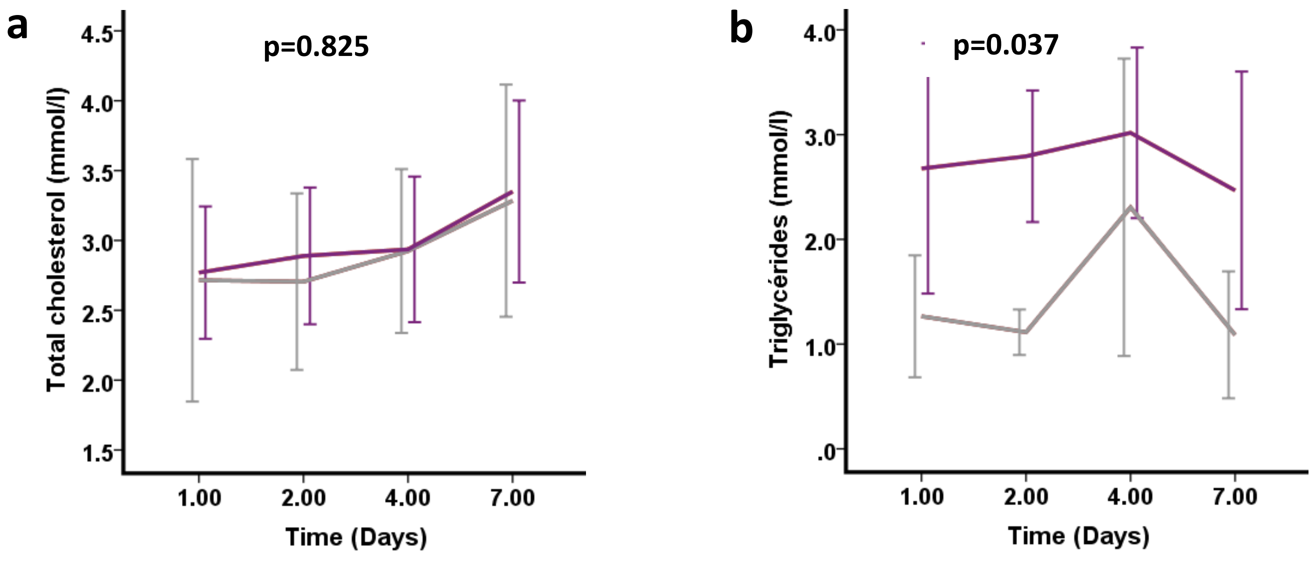

N° of observations				
VA ECMO	8	8	8	3
VV ECMO	17	13	11	7

N° of observations				
VA ECMO	8	8	8	3
VV ECMO	17	13	11	7

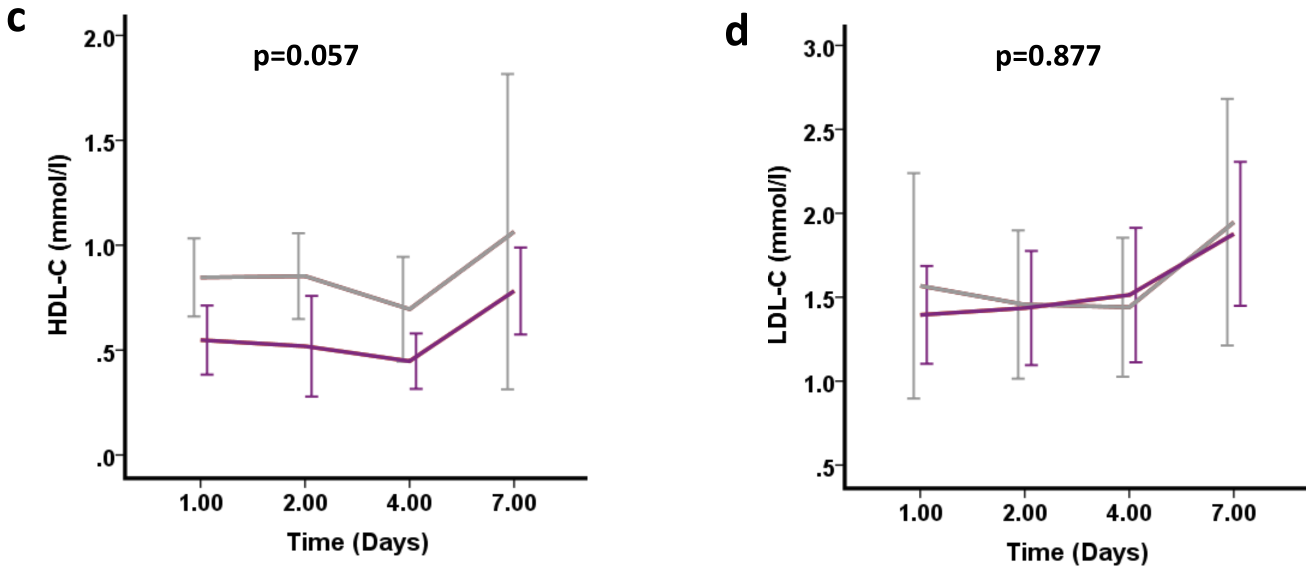

N° of observations				
VA ECMO	8	8	8	3
VV ECMO	17	13	11	7

N° of observations				
VA ECMO	8	8	8	3
VV ECMO	17	13	11	7

Veno-venous ECMO
Veno-arterial ECMO

Supplemental table S3 : correlation between lipoproteins concentrations on the day of ECMO implantation and ECMO duration, mechanical ventilation duration and ICU length of stay.

		**ECMO duration**	**Mechanical ventilation duration**	**Days alive at 28 days without mechanical ventilation**	**ICU length of stay**
**Total cholesterol at ECMO implantation**	Rho of Spearman	-0.228	0.150	-0.126	0.069
	p	0.272	0.474	0.557	0.748
	Number of observations	25	25	25	25
**Triglyceride at ECMO implantation**	Rho of Spearman	0.231	0.104	-0.169	0.147
	p	0.267	0.622	0.429	0.493
	Number of observations	25	25	25	25
**LDL-C at ECMO implantation**	Rho of Spearman	-0.535	-0.076	-0.022	-0.123
	p	0.006	0.718	0.919	0.567
	Number of observations	25	25	24	24
**HDL-C at ECMO implantation**	Rho of Spearman	-0.455	-0.064	0.103	-0.115
	p	0.022	0.760	0.631	0.593
	Number of observations	25	25	25	25
